# Dynamics of SARS-CoV-2 Antibody Response to CoronaVac followed by Booster Dose of BNT162b2 Vaccine

**DOI:** 10.3201/eid2806.220061

**Published:** 2022-06

**Authors:** Marcela Helena Gambim Fonseca, Ana Carolina Matias Dinelly Pinto, Maria Francilene Souza Silva, Amanda Campelo Lima de Melo, Germana Silva Vasconcelos, Eduardo Ruback dos Santos, Fernanda Montenegro de Carvalho Araújo, Luiz Odorico Monteiro de Andrade

**Affiliations:** Fundação Oswaldo Cruz, Eusébio, Brazil

**Keywords:** COVID-19, coronavirus disease, SARS-CoV-2, severe acute respiratory syndrome coronavirus 2, viruses, respiratory infections, zoonoses, vaccine-preventable diseases, CoronaVac, BNT162b2 vaccine, antibody response, healthcare workers

## Abstract

We evaluated the longitudinal dynamics of antibody response to the SARS-CoV-2 vaccine CoronaVac and the effect of a booster dose of BNT162b2 vaccine. We found a robust antibody response after the second dose of CoronaVac that wanes over time. The response was recovered by BNT162b2, which boosted anti-spike antibody titers.

Long-term protection against SARS-CoV-2 requires the persistence of vaccine antibodies above protective thresholds, the maintenance of immune memory cells capable of reactivation after subsequent viral exposure, or both (*1*). A decay of circulating SARS-CoV-2 antibodies over time in persons who received CoronaVac (Sinovac, http://www.sinovac.com) have been reported, suggesting the necessity of a third shot of vaccine (*2*). In Brazil, the third dose has been administered, preferably, with the BNT162b2 vaccine (Pfizer-BioNTech, https://www.pfizer.com) (*3*,*4*). Limited information is available about antibody dynamics after CoronaVac vaccine and the recent supplementing with the BNT162b2 booster. Therefore, we evaluated the longitudinal dynamics of the antibody response to CoronaVac up to 230 days after the second dose in a cohort of healthcare workers (HCWs) and evaluated the effect of a booster dose of BNT162b2 on antibody levels. The study was approved by the Ethics Committee of the Hospital Geral Dr. César Cals (Fortaleza, Brazil; approval no. CAAE 39691420.7.0000.5049). We obtained informed consent from all participants.

## The Study

We included in this study 99 HCWs of both sexes, >18 years of age, who had received 2 doses of the CoronaVac vaccine, with an interval of 28 days between doses, and then a booster shot of BNT162b2 vaccine 8 months after the second CoronaVac dose. Blood collections and serologic tests were performed at Fundação Oswaldo Cruz (Fiocruz; Ceará, Brazil) and analyzed at 7 different timepoints: before vaccination (P1); 28 days after the first CoronaVac dose (P2); 30 (P3) 90 (P4), 180 (P5), and 230 (P6) days after the second CoronaVac dose; and 15 days after the BNT162b2 dose (P7). We monitored the HCWs for SARS-CoV-2 infection by PCR over time.

We tested all serum samples for IgG against nucleocapsid (N) and spike (S) proteins of SARS-CoV-2 by using chemiluminescent microparticle immunoassays on the ARCHITECT i2000SR equipment (Abbott, https://www.abbott.com). The cutoff value was 50 AU/mL for S antibodies and 1.4 index (S/CO) for N antibodies.

We used GraphPad Prism version 9 (https://www.graphpad.com) for statistical analyses. We describe data as median and interquartile range (IQR) or percentage. In group comparisons, we used χ^2^ test to analyze the seropositivity data and Kruskal–Wallis test with subsequent Dunn’s test to analyze the IgG values. We considered differences with p<0.05 to be statistically significant.

The cohort was 70.71% women and 29.29% men. Average age was 32.31 years (95% CI 30.3–34.3 years). The age distribution of persons was as follows: 18–30 years, 42 (42.4%); 31–45 years, 51 (51.5%); and >45 years, 6 (6.1%). Median age for each age group was as follows: 18–30 years, 23.0 years (IQR 20–27.3 years); 31–45 years, 36 years (IQR 32–39 years), >45 years, 56.50 years (IQR 52–63.3 years).

Although all HCWs completed the vaccination schedule, some HCWs were unable to give a blood sample in subsequent phases of the study. Therefore, serum samples were obtained from 99 participants in P1 and P2, 95 in P3, 94 in P4, 89 in P5, 84 in P6, and 74 in P7.

**Figure 1:**
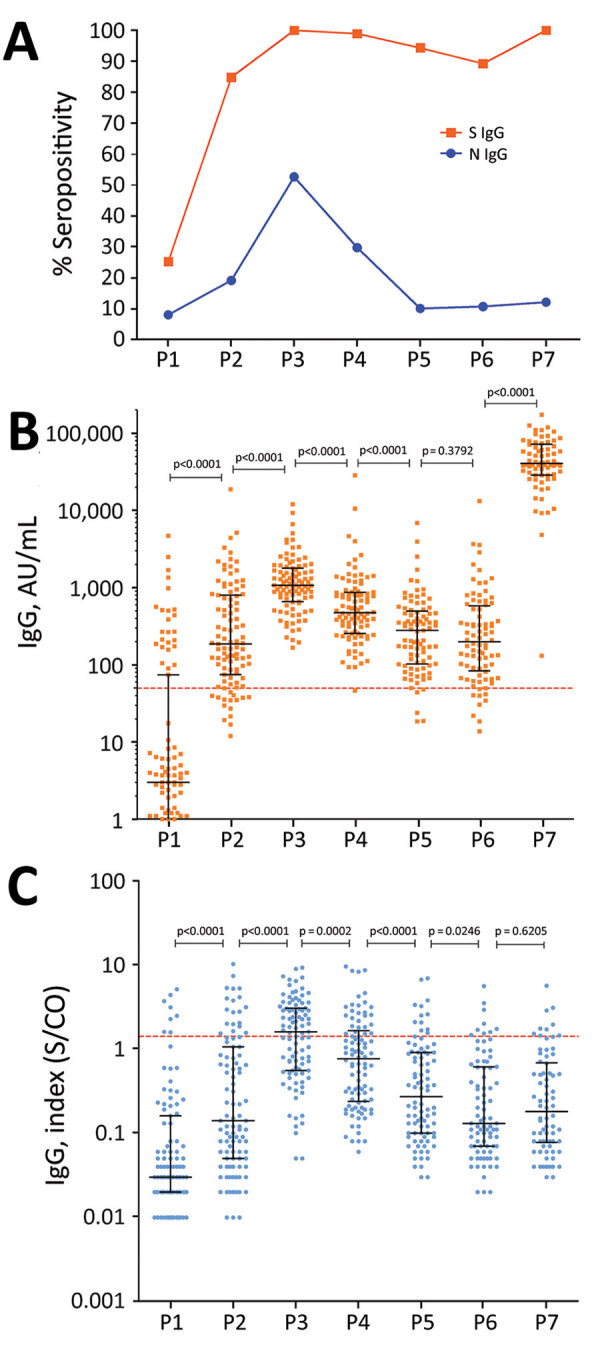
Antibody response over time in a cohort of healthcare workers vaccinated with 2 doses of CoronaVac vaccine (https://www.sinovac.com) followed by a BNT162b2 vaccine (Pfizer-BioNTech, https://www.pfizer.com) booster dose. A) S and N IgG seropositivity. B) S IgG levels. C) N IgG levels. Antibody responses were evaluated before vaccination (timepoint P1); 28 days after the first dose of CoronaVac vaccine (P2); 30 (P3) 90 (P4), 180 (P5), and 230 (P6) days after the second dose of CoronaVac vaccine; and 15 days after the booster dose with BNT162b2 vaccine (P7). For panels B and C, black lines indicate median levels values and error bars interquartile ranges; horizontal dotted lines indicate cutoff values. Statistical analysis performed using the Kruskal–Wallis test with subsequent Dunn’s multiple testing correction. N, nucleocapsid protein; S, spike protein; S/CO, signal-to-cutoff ratio.

We evaluated the seropositivity and IgG levels for S and N proteins at the different timepoints (Figure 1). At baseline (P1), S IgG were detectable in 25.3% of HCWs, increasing to 84.9% in P2 and reaching 100% in P3. We then observed a decline in seropositivity was observed to 98.9% in P4, 94.4% in P5, and 89.3% in P6. In P7, seropositivity had recovered to 100%. N IgG was detectable in 8.1% of HCWs in P1, 19.2% in P2, and 52.6% in P3, then reduced to 29.8% in P4, 13.5% in P5, 10.7% in P6, and 12.2% in P7 (Figure 1, panel A). The seroconversion rate was 79.7% for S antibodies and 11.9% for N antibodies after the first CoronaVac dose, increasing to 100% for S antibodies and 43.6% N antibodies after the second dose.

After the first CoronaVac dose (P2), S IgG levels were significantly elevated compared with baseline values (p<0.0001) (Figure 1, panel B; Appendix Table 1). Those S IgG levels increased significantly after the second dose (P3) (p<0.0001). However, antibodies levels waned over time. The third BNT162b2 dose again significantly increased S IgG levels (p<0.0001). We observed a similar change in N IgG (Figure 1, panel C; Appendix Table 1). Median values of N IgG were significantly higher after the second CoronaVac dose (p<0.0001) and declined significantly after vaccination (p = 0.0002). In contrast, the third dose with BNT162b2 did not increase N IgG levels.

We evaluated the antibody response to the vaccine in relation to a previous SARS-CoV-2 infection. Twenty-five volunteers were seropositive in P1 and were included in the COVID-19–positive group. Eight volunteers had a positive PCR result during the study (4 in P3, 4 in P4); they were moved to the COVID-19–positive group.

**Figure 2:**
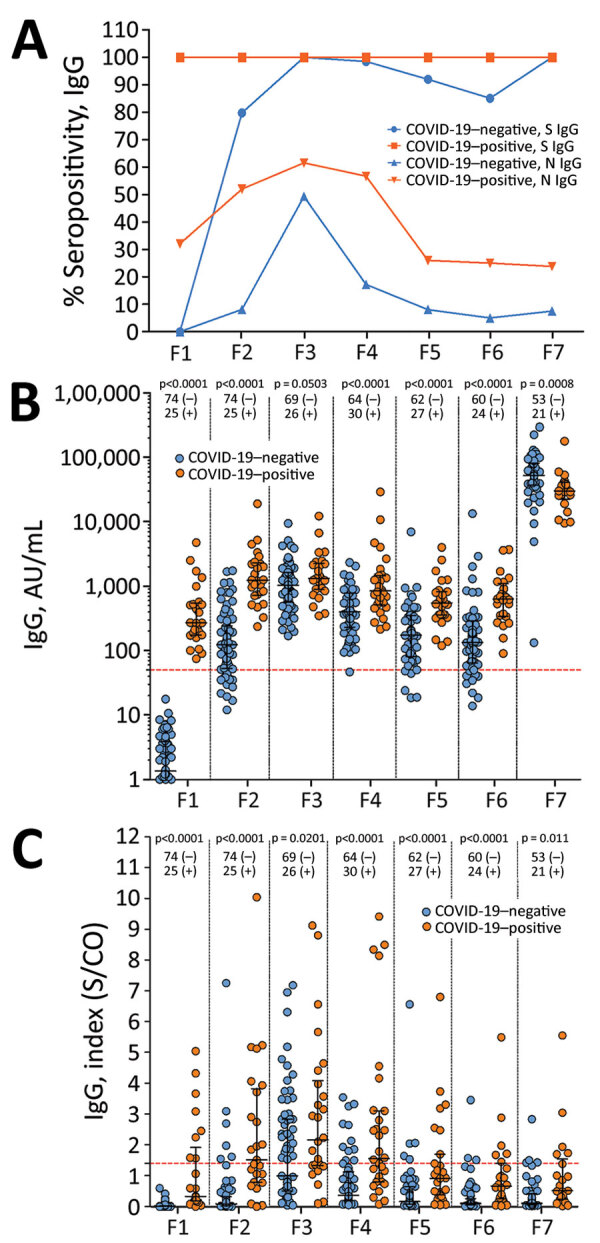
Comparison of antibody response over time among healthcare workers vaccinated with 2 doses of CoronaVac vaccine (https://www.sinovac.com) followed by a BNT162b2 vaccine (Pfizer-BioNTech, https://www.pfizer.com) booster dose, by COVID-19 positivity status. A) S and N IgG seropositivity. B) S IgG levels. C) N IgG levels. Antibody responses were evaluated before vaccination (timepoint P1); 28 days after the first dose of CoronaVac vaccine (P2); 30 (P3) 90 (P4), 180 (P5), and 230 (P6) days after the second dose of CoronaVac vaccine; and 15 days after the booster dose with BNT162b2 vaccine (P7). For panels B and C, black lines indicate median levels values and error bars interquartile ranges; the horizontal dotted line indicates the cutoff value of the assays. Numbers below p values indicate numbers of COVID-19–positive and COVID-19–negative persons in each timepoint. Statistical analysis performed using the Kruskal–Wallis test with subsequent Dunn’s multiple testing correction. N, nucleocapsid protein; S, spike protein; S/CO, signal-to-cutoff ratio; (–), COVID-19 negative; (+), COVID-19 positive.

The HCWs who had COVID-19 maintained the anti-S seropositivity at 100% over time. In relation to HCWs who did not have COVID-19, 79.7% of persons became seropositive to S protein in P2. The seropositivity increased to 100% in P3 but decreased in the next timepoints, recovering in P7 (Figure 2, panel A). The differences in seropositivity between the groups were statistically significant in P2 (p = 0.0145). Anti-N seropositivity was 6 (95% CI 2.7–15.1) times higher in the COVID-19–positive group in P2 compared with the COVID-19–negative group. This difference was reduced in P3, increasing in P4 and stabilizing in P5, reaching levels of 5 (95% CI 1.4–18.4) times higher in the COVID-19–positive group.

In the antibody levels analysis, antibody titers for S protein were higher in the COVID-19–positive group than for the COVID-19–negative group at all timepoints except P3 and P7 (Figure 2, panel B; Appendix Table 2). N IgG levels of COVID-19–positive persons were statistically higher than for COVID-19–negative persons at all timepoints (Figure 2, panel C; Appendix Table 2).

## Conclusions

We found an antibody response to N and S protein after 2 doses of CoronaVac vaccine. However, the antibodies declined over time. After immunization, the decline of antibodies is expected because not all vaccine-induced plasmablasts commit or are maintained as long-lived memory plasma cells (*5*). Thus, the success of vaccines depends on the generation and maintenance of immunologic memory (*6*). Administration of BNT162b2 as the third vaccine dose boosted S IgG but not N IgG. The substantial increase of S IgG after the booster dose suggests that CoronaVac vaccine induced immune memory. The third BNT162b2 dose did not increase the N IgG because mRNA vaccines do not induce a response to the N protein (*7*,*8*).

Previously infected participants had a significantly higher antibody level than previously uninfected participants in almost all phases of the study. In addition, we found that those without previous infection showed a faster waning of antibodies over time, a result also reported in previous studies (*9*). The antibody-making B cells multiply after each exposure, whether attributable to the infection or vaccination; therefore, antibody levels in the previously infected HCWs can reflect the sum of the antibodies produced after infection and vaccine (*10*).

In summary, a booster dose of BNT162b2 vaccine in HCWs administered 8 months after the second dose with CoronaVac vaccine recalled a specific immune response to SARS-CoV-2. That response had declined substantially 230 days after the second dose of CoronaVac vaccine, resulting in an increase of S IgG after BNT162b2 vaccination and indicating that the 2-dose CoronaVac vaccine schedule generates immune memory.

Appendix Additional information about dynamics of SARS-CoV-2 antibody response to CoronaVac followed by booster dose of BNT162b2 vaccine.
