# Supplementary material for: Dynamics of SARS-CoV-2 Antibody Response to CoronaVac followed by Booster Dose of BNT162b2 Vaccine
Source: Emerg Infect Dis. 2022 Jun;28(6):1237–40. doi: 10.3201/eid2806.220061 (PMC9155880; doi:10.3201/eid2806.220061)
Supplement: Appendix — Additional information about dynamics of SARS-CoV-2 antibody response to CoronaVac followed by booster dose of BNT162b2 vaccine. [file 22-0061-Techapp-s1.pdf]

Article DOI: https://doi.org/10.3201/eid2806.220061

# Dynamics of SARS-CoV-2 Antibody Response to CoronaVac followed by Booster Dose of BNT162b2 Vaccine

## Appendix

**Appendix Table 1.** The median (and IQR) values of the antibody levels in response to CoronaVac and BNT162b2 vaccines

| | Median AU/mL (IQR) | |
|---|---|---|
| Timepoint, no. participants | **S IgG antibodies** | **N IgG antibodies** |
| P1, n = 99 | 3.0 (0.9–74.70) | 0.03 (0.02–0.16) |
| P2, n = 99 | 188.0 (75–806.5) | 0.14 (0.05–1.05) |
| P3, n = 95 | 1,081 (665–1,811) | 1.58 (0.55–3.01) |
| P4, n = 94 | 477.7 (256.8–880.2) | 0.76 (0.24–1.63) |
| P5, n = 89 | 282.3 (103.1–500.5) | 0.27 (0.10–0.90) |
| P6, n = 84 | 200.2 (84.18–585.7) | 0.13 (0.07–0.61) |
| P7, n = 74 | 41,371 (29,233–73,465) | 0.18 (0.08–0.68) |

**Appendix Table 2.** The median (and IQR) values of the antibody levels in response to CoronaVac and BNT162b2 vaccines in COVID-19 positive and negative persons

| Timepoint, no. | COVID-19 negative | | COVID-19 positive | |
|---|---|---|---|---|
| participants | **S IgG antibodies** | **N IgG antibodies** | **S IgG antibodies** | **N IgG antibodies** |
| P1, n = 99 | 1.35 (0.55–3.83) | 0.03 (0.02–0.04) | 270.2 (173.5–548.2) | 0.32 (0.18–1.91) |
| P2, n = 99 | 124.1 (52.98–242) | 0.09 (0.04–0.32) | 1,245 (725.3–2,296) | 1.51 (0.78–3.82) |
| P3, n = 95 | 1,035 (563- 1,628) | 0.99 (0.51–2.83) | 1,323 (875–2,263) | 2.17 (1.32–4.08) |
| P4, n = 94 | 399 (231.2–777.5) | 0.36 (0.19–1.11) | 845.8 (509.2–1,462) | 1.55 (0.80–3.11) |
| P5, n = 89 | 172.9 (80.53–349) | 0.16 (0.09–0.65) | 546.4 (356.6–724.9) | 0.91 (0.37–1.69) |
| P6, n = 84 | 133.5 (64.83–289.4) | 0.11 (0.06–0.25) | 627.4 (342.6–1,098) | 0.66 (0.25–1.40) |
| P7, n = 74 | 52,372 (37,075–81,365) | 0.11 (0.06–0.41) | 30,067 (22,276–40,679) | 0.51 (0.23–1.54) |
